# Bis(μ-*N*-nitroso-*N*-phenyl­hydroxy­laminato)-κ^3^ *O*,*O*′:*O*′;κ^3^ *O*′:*O*,*O*′-bis­[(*N*-nitroso-*N*-phenyl­hydroxy­laminato-κ^2^ *O*,*O*′)lead(II)]

**DOI:** 10.1107/S1600536811006775

**Published:** 2011-02-26

**Authors:** Ezzatollah Najafi, Mostafa M. Amini, Seik Weng Ng

**Affiliations:** a Department of Chemistry, General Campus, Shahid Beheshti University, Tehran 1983963113, Iran; b Department of Chemistry, University of Malaya, 50603 Kuala Lumpur, Malaysia

## Abstract

The four cupferronate ions in the dinuclear title compound, [Pb_2_(C_6_H_5_N_2_O_2_)_4_], *O*,*O*′-chelate to the two Pb^II^ atoms; two of the four nitroso O atoms are also involved in bridging. The geometry of both five-coordinate Pb^II^atoms is distorted Ψ-octa­hedral; if another two longer inter­molecular Pb⋯O inter­actions [at 2.955 (1) and 3.099 (1) Å] are considered, the geometry is a distorted Ψ-square anti­prism.

## Related literature

For the spectrospic assigment of the structure of the lead derivative, see: Bottei & Schneggenburger (1970 ▶). For the structure of the organic ligand, see: Hickmann *et al.* (1979 ▶).
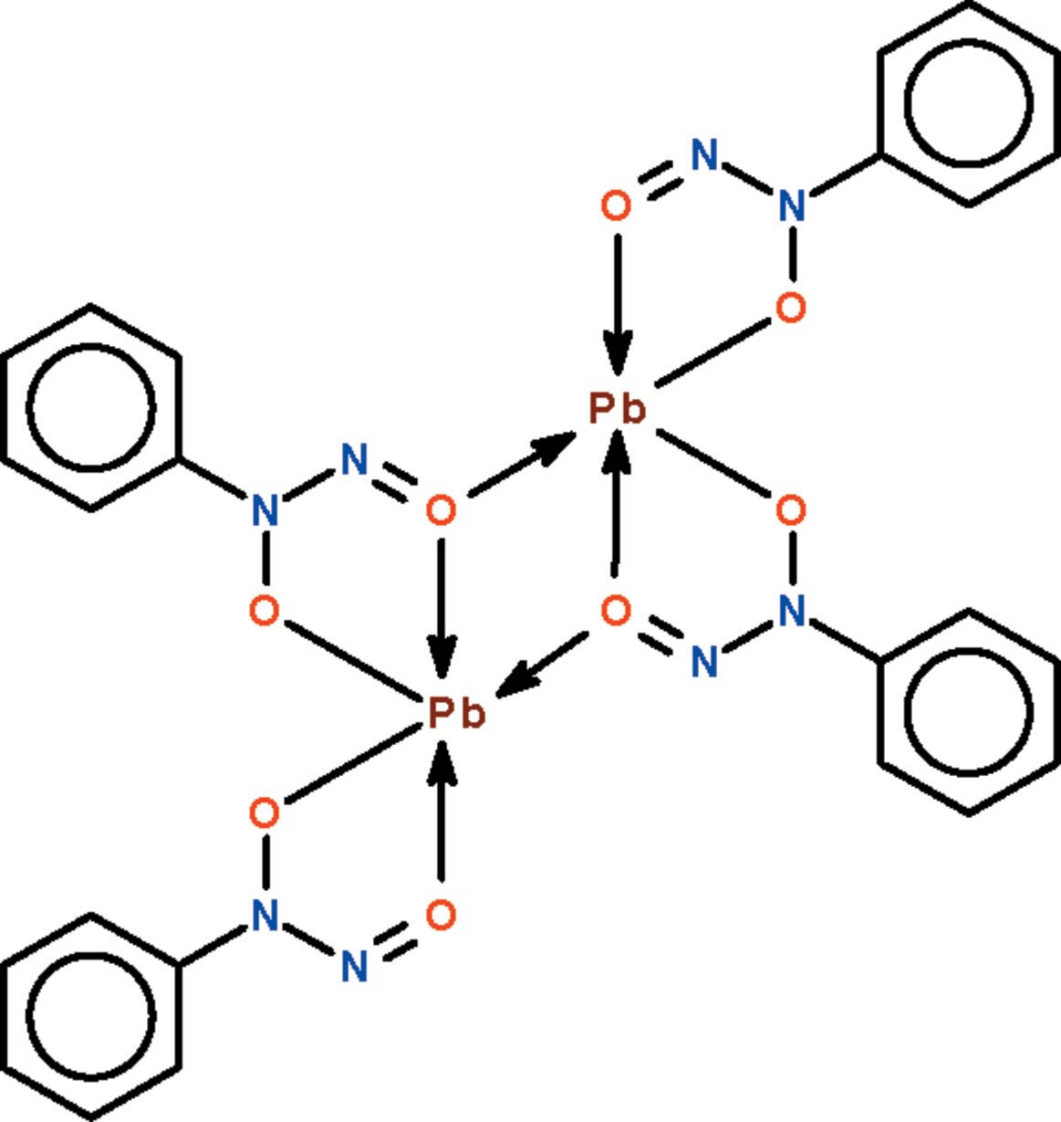


## Experimental

### 

#### Crystal data

[Pb_2_(C_6_H_5_N_2_O_2_)_4_]
*M* *_r_* = 962.86
Triclinic, 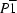

*a* = 9.6149 (5) Å
*b* = 11.5340 (6) Å
*c* = 13.2724 (7) Å
α = 82.459 (1)°
β = 79.280 (1)°
γ = 67.369 (1)°
*V* = 1331.95 (12) Å^3^
*Z* = 2
Mo *K*α radiation
μ = 12.69 mm^−1^
*T* = 100 K
0.30 × 0.15 × 0.15 mm

#### Data collection

Bruker SMART APEX diffractometer
Absorption correction: multi-scan (*SADABS*; Sheldrick, 1996 ▶) *T*_min_ = 0.115, *T*_max_ = 0.252
16878 measured reflections
6094 independent reflections
5444 reflections with *I* > 2σ(*I*)
*R*_int_ = 0.036

#### Refinement

*R*[*F* ^2^ > 2σ(*F* ^2^)] = 0.026
*wR*(*F* ^2^) = 0.071
*S* = 1.06
6094 reflections
379 parameters
H-atom parameters constrained
Δρ_max_ = 1.74 e Å^−3^
Δρ_min_ = −2.24 e Å^−3^

### 

Data collection: *APEX2* (Bruker, 2009 ▶); cell refinement: *SAINT* (Bruker, 2009 ▶); data reduction: *SAINT*; program(s) used to solve structure: *SHELXS97* (Sheldrick, 2008 ▶); program(s) used to refine structure: *SHELXL97* (Sheldrick, 2008 ▶); molecular graphics: *X-SEED* (Barbour, 2001 ▶); software used to prepare material for publication: *publCIF* (Westrip, 2010 ▶).

## Supplementary Material

Crystal structure: contains datablocks global, I. DOI: 10.1107/S1600536811006775/bt5480sup1.cif

Structure factors: contains datablocks I. DOI: 10.1107/S1600536811006775/bt5480Isup2.hkl

Additional supplementary materials:  crystallographic information; 3D view; checkCIF report

## Figures and Tables

**Fig. 1.**
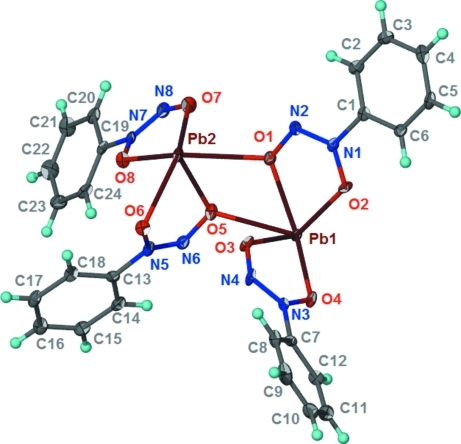
Anisotropic displacement ellipsoid plot (Barbour, 2001) of Pb2(C6H5N2O2)4 at the 70% probability level. Hydrogen atoms are drawn as spheres of arbitrary radius.

**Fig. 2.**
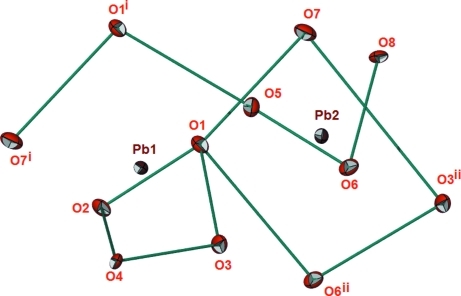
Detail of geometry of the lead atoms when intermolecular longer interactions are considered. Symmetry codes: (i) 1 - x, 1 - y, 1 - z; (ii) 2 - x, 1 - y, 1 - z.

## supplementary crystallographic information

### Comment

The cupferronate ion is a common ion used for the complexation of metals; the crystal structure of the chelate has been reported (Hickmann *et al.*, 1979). The synthesis of the lead(II) derivative has been known for a long time (Bottei & Schneggenburger, 1970), and the compound was assumed to exist as a mononuclear compound. The compound is, in fact, a dinuclear compound (Scheme I). The four cupferronate ions in dinuclear [Pb(C_6_H_5_N_2_O_2_)_2_]_2_*O*,*O*'-chelate to the lead(II) atom; two of the four nitroso O atoms are also involved in bridging (Fig. 1). The geometry of both five-coordinate lead atoms is Ψ-octahedral; if another longer intermolecular Pb···O interactions (approx. 3.0 Å) are considered, the geometry is a Ψ-square-antiprism (Fig. 2).

### Experimental

Lead(II) nitrate (0.33 g, 1 mmol) dissolved in ethanol (20 ml) was added to the cupferron ligand (0.31 g, 2 mmol) dissolved in ethanol (20 ml). The mixture was stirred and then set aside for the growth of brown colored crystals.

### Refinement

Hydrogen atoms were placed in calculated positions (C–H 0.95 Å) and were included in the refinement in the riding model approximation, with *U*(H) set to 1.2*U*_eq_(C).

Omitted from the refinement were the following reflections owing to bad disagreement between the observed and calculated *F*^2^ values: (0 0 1), (0 1 2), (1 0 1), (0 0 2), (11 4 7), (-9 - 11 5), (11 3 8), (11 5 6), (-4 - 9 10), (-9 -9 2) and (-4 7 0).  The final difference Fourier map had a peak in the vicinity of Pb2 and a hole in the vicinty of the same atom.

### Crystal data

**Table:**

[Pb_2_(C_6_H_5_N_2_O_2_)_4_]	*Z* = 2
*M**_r_* = 962.86	*F*(000) = 896
Triclinic, *P*1	*D*_x_ = 2.401 Mg m^−^^3^
Hall symbol: -P 1	Mo *K*α radiation, λ = 0.71073 Å
*a* = 9.6149 (5) Å	Cell parameters from 9907 reflections
*b* = 11.5340 (6) Å	θ = 2.4–28.3°
*c* = 13.2724 (7) Å	µ = 12.69 mm^−^^1^
α = 82.459 (1)°	*T* = 100 K
β = 79.280 (1)°	Prism, brown
γ = 67.369 (1)°	0.30 × 0.15 × 0.15 mm
*V* = 1331.95 (12) Å^3^	

### Data collection

**Table:**

Bruker SMART APEX diffractometer	6094 independent reflections
Radiation source: fine-focus sealed tube	5444 reflections with *I* > 2σ(*I*)
graphite	*R*_int_ = 0.036
ω scans	θ_max_ = 27.5°, θ_min_ = 2.3°
Absorption correction: multi-scan (*SADABS*; Sheldrick, 1996)	*h* = −12→12
*T*_min_ = 0.115, *T*_max_ = 0.252	*k* = −14→14
16878 measured reflections	*l* = −17→17

### Refinement

**Table:**

Refinement on *F*^2^	Primary atom site location: structure-invariant direct methods
Least-squares matrix: full	Secondary atom site location: difference Fourier map
*R*[*F*^2^ > 2σ(*F*^2^)] = 0.026	Hydrogen site location: inferred from neighbouring sites
*wR*(*F*^2^) = 0.071	H-atom parameters constrained
*S* = 1.06	*w* = 1/[σ^2^(*F*_o_^2^) + (0.0339*P*)^2^ + 2.8506*P*] where *P* = (*F*_o_^2^ + 2*F*_c_^2^)/3
6094 reflections	(Δ/σ)_max_ = 0.001
379 parameters	Δρ_max_ = 1.74 e Å^−^^3^
0 restraints	Δρ_min_ = −2.24 e Å^−^^3^

### Fractional atomic coordinates and isotropic or equivalent isotropic displacement parameters (Å^2^)

**Table:**

	*x*	*y*	*z*	*U*_iso_*/*U*_eq_	
Pb1	0.634932 (18)	0.462439 (15)	0.364998 (12)	0.00867 (6)	
Pb2	0.819969 (18)	0.523384 (15)	0.612158 (13)	0.01006 (6)	
O1	0.6060 (4)	0.6161 (3)	0.4843 (2)	0.0123 (7)	
O2	0.5388 (4)	0.6773 (3)	0.3020 (2)	0.0121 (7)	
O3	0.8865 (4)	0.4622 (3)	0.3460 (3)	0.0151 (7)	
O4	0.7942 (4)	0.3974 (3)	0.2021 (3)	0.0120 (7)	
O5	0.7358 (4)	0.3785 (3)	0.5523 (3)	0.0143 (7)	
O6	1.0141 (4)	0.3240 (3)	0.5670 (3)	0.0134 (7)	
O7	0.5918 (4)	0.5665 (3)	0.7423 (3)	0.0163 (7)	
O8	0.8387 (4)	0.3773 (3)	0.7572 (3)	0.0131 (7)	
N1	0.5102 (4)	0.7608 (4)	0.3685 (3)	0.0105 (8)	
N2	0.5430 (5)	0.7366 (4)	0.4610 (3)	0.0135 (8)	
N3	0.9326 (4)	0.3966 (4)	0.1922 (3)	0.0083 (7)	
N4	0.9843 (5)	0.4281 (4)	0.2626 (3)	0.0125 (8)	
N5	0.9547 (5)	0.2375 (4)	0.5810 (3)	0.0113 (8)	
N6	0.8147 (4)	0.2585 (4)	0.5742 (3)	0.0124 (8)	
N7	0.7129 (4)	0.3892 (4)	0.8210 (3)	0.0108 (8)	
N8	0.5862 (5)	0.4818 (4)	0.8174 (3)	0.0160 (9)	
C1	0.4389 (5)	0.8924 (4)	0.3363 (4)	0.0100 (9)	
C2	0.3808 (5)	0.9821 (5)	0.4097 (4)	0.0135 (9)	
H2	0.3868	0.9575	0.4804	0.016*	
C3	0.3142 (6)	1.1081 (5)	0.3774 (4)	0.0155 (10)	
H3	0.2736	1.1703	0.4264	0.019*	
C4	0.3064 (6)	1.1439 (5)	0.2730 (4)	0.0160 (10)	
H4	0.2630	1.2304	0.2509	0.019*	
C5	0.3626 (6)	1.0524 (5)	0.2020 (4)	0.0154 (10)	
H5	0.3563	1.0766	0.1313	0.018*	
C6	0.4280 (5)	0.9255 (4)	0.2332 (4)	0.0116 (9)	
H6	0.4644	0.8630	0.1846	0.014*	
C7	1.0322 (5)	0.3611 (4)	0.0962 (3)	0.0093 (9)	
C8	1.1523 (5)	0.4028 (5)	0.0675 (4)	0.0142 (10)	
H8	1.1735	0.4506	0.1116	0.017*	
C9	1.2405 (6)	0.3726 (5)	−0.0276 (4)	0.0208 (11)	
H9	1.3242	0.3991	−0.0488	0.025*	
C10	1.2081 (6)	0.3047 (5)	−0.0917 (4)	0.0173 (10)	
H10	1.2680	0.2866	−0.1573	0.021*	
C11	1.0883 (6)	0.2625 (5)	−0.0609 (4)	0.0179 (10)	
H11	1.0678	0.2140	−0.1047	0.021*	
C12	0.9989 (5)	0.2915 (4)	0.0341 (4)	0.0130 (9)	
H12	0.9163	0.2638	0.0558	0.016*	
C13	1.0454 (5)	0.1123 (4)	0.6183 (4)	0.0114 (9)	
C14	0.9955 (6)	0.0140 (5)	0.6234 (4)	0.0156 (10)	
H14	0.9035	0.0261	0.5994	0.019*	
C15	1.0836 (6)	−0.1037 (5)	0.6649 (4)	0.0160 (10)	
H15	1.0517	−0.1728	0.6689	0.019*	
C16	1.2180 (6)	−0.1200 (5)	0.7002 (4)	0.0164 (10)	
H16	1.2769	−0.2001	0.7287	0.020*	
C17	1.2661 (6)	−0.0205 (5)	0.6942 (4)	0.0147 (10)	
H17	1.3585	−0.0326	0.7179	0.018*	
C18	1.1794 (5)	0.0983 (5)	0.6533 (4)	0.0123 (9)	
H18	1.2110	0.1675	0.6495	0.015*	
C19	0.7167 (5)	0.2865 (4)	0.8969 (4)	0.0102 (9)	
C20	0.6247 (6)	0.3103 (5)	0.9917 (4)	0.0151 (10)	
H20	0.5615	0.3939	1.0083	0.018*	
C21	0.6273 (6)	0.2085 (5)	1.0620 (4)	0.0191 (11)	
H21	0.5645	0.2225	1.1270	0.023*	
C22	0.7215 (6)	0.0868 (5)	1.0372 (4)	0.0217 (11)	
H22	0.7216	0.0175	1.0847	0.026*	
C23	0.8148 (6)	0.0666 (5)	0.9434 (4)	0.0180 (10)	
H23	0.8806	−0.0166	0.9275	0.022*	
C24	0.8139 (6)	0.1657 (5)	0.8724 (4)	0.0151 (10)	
H24	0.8785	0.1515	0.8081	0.018*	

### Atomic displacement parameters (Å^2^)

**Table:**

	*U*^11^	*U*^22^	*U*^33^	*U*^12^	*U*^13^	*U*^23^
Pb1	0.00879 (9)	0.01181 (9)	0.00641 (9)	−0.00508 (7)	−0.00091 (6)	−0.00028 (6)
Pb2	0.01043 (9)	0.01290 (10)	0.00908 (10)	−0.00683 (7)	−0.00345 (7)	0.00231 (7)
O1	0.0151 (17)	0.0133 (16)	0.0089 (16)	−0.0059 (14)	−0.0030 (13)	0.0017 (13)
O2	0.0169 (17)	0.0132 (16)	0.0069 (15)	−0.0060 (13)	−0.0007 (13)	−0.0032 (13)
O3	0.0140 (17)	0.0257 (19)	0.0091 (16)	−0.0100 (15)	−0.0017 (13)	−0.0051 (14)
O4	0.0080 (15)	0.0203 (17)	0.0103 (16)	−0.0077 (13)	−0.0027 (12)	−0.0005 (13)
O5	0.0142 (17)	0.0167 (17)	0.0143 (17)	−0.0077 (14)	−0.0058 (14)	0.0022 (14)
O6	0.0122 (16)	0.0150 (16)	0.0155 (17)	−0.0084 (13)	−0.0013 (13)	0.0009 (13)
O7	0.0172 (18)	0.0186 (18)	0.0096 (17)	−0.0051 (14)	0.0011 (14)	0.0016 (14)
O8	0.0081 (15)	0.0190 (17)	0.0104 (16)	−0.0053 (13)	0.0017 (12)	0.0015 (13)
N1	0.0095 (18)	0.0153 (19)	0.0080 (19)	−0.0064 (16)	−0.0009 (15)	−0.0004 (15)
N2	0.013 (2)	0.014 (2)	0.013 (2)	−0.0046 (16)	−0.0042 (16)	0.0004 (16)
N3	0.0049 (17)	0.0130 (18)	0.0070 (18)	−0.0045 (15)	0.0010 (14)	0.0004 (15)
N4	0.0120 (19)	0.022 (2)	0.0062 (19)	−0.0099 (17)	−0.0033 (15)	0.0020 (16)
N5	0.0138 (19)	0.016 (2)	0.0060 (18)	−0.0087 (16)	−0.0020 (15)	0.0022 (15)
N6	0.0101 (19)	0.015 (2)	0.014 (2)	−0.0058 (16)	−0.0024 (15)	−0.0025 (16)
N7	0.0113 (19)	0.0161 (19)	0.0047 (18)	−0.0047 (16)	−0.0021 (14)	0.0000 (15)
N8	0.019 (2)	0.019 (2)	0.0083 (19)	−0.0047 (17)	−0.0031 (16)	0.0006 (16)
C1	0.007 (2)	0.013 (2)	0.011 (2)	−0.0056 (17)	−0.0005 (17)	0.0002 (17)
C2	0.012 (2)	0.019 (2)	0.010 (2)	−0.0079 (19)	0.0031 (18)	−0.0032 (18)
C3	0.014 (2)	0.017 (2)	0.016 (2)	−0.0059 (19)	−0.0004 (19)	−0.006 (2)
C4	0.014 (2)	0.015 (2)	0.022 (3)	−0.0094 (19)	−0.008 (2)	0.006 (2)
C5	0.017 (2)	0.022 (3)	0.009 (2)	−0.008 (2)	−0.0055 (18)	0.0035 (19)
C6	0.011 (2)	0.013 (2)	0.012 (2)	−0.0057 (18)	−0.0021 (18)	−0.0023 (18)
C7	0.010 (2)	0.008 (2)	0.007 (2)	−0.0012 (17)	0.0019 (17)	−0.0004 (16)
C8	0.010 (2)	0.018 (2)	0.016 (2)	−0.0072 (19)	−0.0045 (18)	0.0034 (19)
C9	0.010 (2)	0.027 (3)	0.024 (3)	−0.008 (2)	−0.004 (2)	0.008 (2)
C10	0.015 (2)	0.018 (2)	0.014 (2)	−0.003 (2)	0.0000 (19)	0.001 (2)
C11	0.013 (2)	0.022 (3)	0.017 (3)	−0.003 (2)	−0.0014 (19)	−0.004 (2)
C12	0.010 (2)	0.016 (2)	0.014 (2)	−0.0075 (18)	0.0027 (18)	0.0010 (18)
C13	0.011 (2)	0.013 (2)	0.008 (2)	−0.0029 (18)	0.0016 (17)	−0.0024 (17)
C14	0.015 (2)	0.022 (3)	0.012 (2)	−0.008 (2)	−0.0017 (19)	−0.0048 (19)
C15	0.019 (2)	0.017 (2)	0.017 (2)	−0.011 (2)	−0.003 (2)	−0.0024 (19)
C16	0.014 (2)	0.021 (3)	0.015 (2)	−0.008 (2)	−0.0030 (19)	0.001 (2)
C17	0.013 (2)	0.024 (3)	0.010 (2)	−0.010 (2)	−0.0018 (18)	0.0005 (19)
C18	0.010 (2)	0.019 (2)	0.012 (2)	−0.0111 (19)	0.0003 (18)	−0.0008 (19)
C19	0.011 (2)	0.016 (2)	0.008 (2)	−0.0091 (18)	−0.0026 (17)	−0.0006 (18)
C20	0.015 (2)	0.022 (3)	0.009 (2)	−0.008 (2)	−0.0026 (18)	−0.0011 (19)
C21	0.013 (2)	0.029 (3)	0.015 (3)	−0.010 (2)	0.0030 (19)	0.003 (2)
C22	0.028 (3)	0.019 (3)	0.021 (3)	−0.015 (2)	−0.006 (2)	0.008 (2)
C23	0.024 (3)	0.014 (2)	0.017 (3)	−0.007 (2)	−0.003 (2)	−0.0024 (19)
C24	0.017 (2)	0.021 (2)	0.010 (2)	−0.010 (2)	−0.0013 (19)	−0.0040 (19)

### Geometric parameters (Å, °)

**Table:**

Pb1—O2	2.382 (3)	C5—H5	0.9500
Pb1—O3	2.384 (3)	C6—H6	0.9500
Pb1—O4	2.427 (3)	C7—C12	1.378 (7)
Pb1—O1	2.433 (3)	C7—C8	1.388 (7)
Pb1—O5	2.757 (3)	C8—C9	1.387 (7)
Pb2—O8	2.371 (3)	C8—H8	0.9500
Pb2—O5	2.389 (3)	C9—C10	1.376 (8)
Pb2—O6	2.403 (3)	C9—H9	0.9500
Pb2—O7	2.453 (3)	C10—C11	1.392 (7)
Pb2—O1	2.718 (3)	C10—H10	0.9500
O1—N2	1.305 (5)	C11—C12	1.387 (7)
O2—N1	1.309 (5)	C11—H11	0.9500
O3—N4	1.307 (5)	C12—H12	0.9500
O4—N3	1.310 (5)	C13—C14	1.380 (7)
O5—N6	1.320 (5)	C13—C18	1.394 (7)
O6—N5	1.307 (5)	C14—C15	1.399 (7)
O7—N8	1.311 (5)	C14—H14	0.9500
O8—N7	1.312 (5)	C15—C16	1.393 (7)
N1—N2	1.293 (6)	C15—H15	0.9500
N1—C1	1.447 (6)	C16—C17	1.382 (7)
N3—N4	1.286 (6)	C16—H16	0.9500
N3—C7	1.445 (6)	C17—C18	1.400 (7)
N5—N6	1.291 (6)	C17—H17	0.9500
N5—C13	1.447 (6)	C18—H18	0.9500
N7—N8	1.278 (6)	C19—C24	1.387 (7)
N7—C19	1.444 (6)	C19—C20	1.390 (7)
C1—C6	1.383 (7)	C20—C21	1.396 (7)
C1—C2	1.396 (7)	C20—H20	0.9500
C2—C3	1.388 (7)	C21—C22	1.390 (8)
C2—H2	0.9500	C21—H21	0.9500
C3—C4	1.400 (7)	C22—C23	1.382 (8)
C3—H3	0.9500	C22—H22	0.9500
C4—C5	1.388 (7)	C23—C24	1.381 (7)
C4—H4	0.9500	C23—H23	0.9500
C5—C6	1.392 (7)	C24—H24	0.9500
			
O2—Pb1—O3	91.40 (12)	C4—C5—C6	120.8 (4)
O2—Pb1—O4	92.26 (11)	C4—C5—H5	119.6
O3—Pb1—O4	64.06 (11)	C6—C5—H5	119.6
O2—Pb1—O1	64.21 (11)	C1—C6—C5	118.7 (4)
O3—Pb1—O1	77.66 (11)	C1—C6—H6	120.6
O4—Pb1—O1	134.66 (11)	C5—C6—H6	120.6
O2—Pb1—O5	125.02 (11)	C12—C7—C8	122.4 (4)
O3—Pb1—O5	72.98 (11)	C12—C7—N3	118.0 (4)
O4—Pb1—O5	123.21 (10)	C8—C7—N3	119.5 (4)
O1—Pb1—O5	61.03 (10)	C9—C8—C7	117.9 (5)
O8—Pb2—O5	79.95 (12)	C9—C8—H8	121.0
O8—Pb2—O6	70.65 (11)	C7—C8—H8	121.0
O5—Pb2—O6	64.08 (11)	C10—C9—C8	120.8 (5)
O8—Pb2—O7	63.85 (11)	C10—C9—H9	119.6
O5—Pb2—O7	85.48 (12)	C8—C9—H9	119.6
O6—Pb2—O7	128.79 (11)	C9—C10—C11	120.4 (5)
O8—Pb2—O1	130.44 (11)	C9—C10—H10	119.8
O5—Pb2—O1	62.13 (11)	C11—C10—H10	119.8
O6—Pb2—O1	113.71 (11)	C12—C11—C10	119.7 (5)
O7—Pb2—O1	81.54 (11)	C12—C11—H11	120.1
N2—O1—Pb1	121.0 (3)	C10—C11—H11	120.1
N2—O1—Pb2	121.7 (3)	C7—C12—C11	118.8 (4)
Pb1—O1—Pb2	111.54 (12)	C7—C12—H12	120.6
N1—O2—Pb1	116.1 (3)	C11—C12—H12	120.6
N4—O3—Pb1	122.2 (3)	C14—C13—C18	122.3 (5)
N3—O4—Pb1	115.8 (3)	C14—C13—N5	120.7 (4)
N6—O5—Pb2	115.1 (3)	C18—C13—N5	116.9 (4)
N6—O5—Pb1	121.7 (3)	C13—C14—C15	118.5 (5)
Pb2—O5—Pb1	111.59 (12)	C13—C14—H14	120.8
N5—O6—Pb2	109.6 (3)	C15—C14—H14	120.8
N8—O7—Pb2	119.6 (3)	C16—C15—C14	120.2 (5)
N7—O8—Pb2	117.1 (3)	C16—C15—H15	119.9
N2—N1—O2	125.6 (4)	C14—C15—H15	119.9
N2—N1—C1	116.3 (4)	C17—C16—C15	120.4 (5)
O2—N1—C1	118.0 (4)	C17—C16—H16	119.8
N1—N2—O1	112.5 (4)	C15—C16—H16	119.8
N4—N3—O4	123.6 (4)	C16—C17—C18	120.2 (5)
N4—N3—C7	117.9 (4)	C16—C17—H17	119.9
O4—N3—C7	118.5 (4)	C18—C17—H17	119.9
N3—N4—O3	114.4 (4)	C13—C18—C17	118.3 (5)
N6—N5—O6	124.4 (4)	C13—C18—H18	120.9
N6—N5—C13	116.9 (4)	C17—C18—H18	120.9
O6—N5—C13	118.3 (4)	C24—C19—C20	121.8 (5)
N5—N6—O5	112.6 (4)	C24—C19—N7	118.3 (4)
N8—N7—O8	124.7 (4)	C20—C19—N7	119.9 (4)
N8—N7—C19	118.0 (4)	C19—C20—C21	118.5 (5)
O8—N7—C19	117.1 (4)	C19—C20—H20	120.8
N7—N8—O7	113.5 (4)	C21—C20—H20	120.8
C6—C1—C2	121.7 (4)	C22—C21—C20	120.2 (5)
C6—C1—N1	118.8 (4)	C22—C21—H21	119.9
C2—C1—N1	119.5 (4)	C20—C21—H21	119.9
C3—C2—C1	118.8 (5)	C23—C22—C21	119.9 (5)
C3—C2—H2	120.6	C23—C22—H22	120.0
C1—C2—H2	120.6	C21—C22—H22	120.0
C2—C3—C4	120.4 (5)	C24—C23—C22	121.0 (5)
C2—C3—H3	119.8	C24—C23—H23	119.5
C4—C3—H3	119.8	C22—C23—H23	119.5
C5—C4—C3	119.6 (5)	C23—C24—C19	118.6 (5)
C5—C4—H4	120.2	C23—C24—H24	120.7
C3—C4—H4	120.2	C19—C24—H24	120.7
			
O2—Pb1—O1—N2	5.5 (3)	Pb1—O4—N3—C7	−178.8 (3)
O3—Pb1—O1—N2	103.2 (3)	O4—N3—N4—O3	−0.4 (6)
O4—Pb1—O1—N2	71.0 (4)	C7—N3—N4—O3	178.2 (4)
O5—Pb1—O1—N2	−179.6 (3)	Pb1—O3—N4—N3	0.8 (5)
O2—Pb1—O1—Pb2	−148.12 (16)	Pb2—O6—N5—N6	28.7 (5)
O3—Pb1—O1—Pb2	−50.45 (13)	Pb2—O6—N5—C13	−143.8 (3)
O4—Pb1—O1—Pb2	−82.70 (17)	O6—N5—N6—O5	−0.8 (6)
O5—Pb1—O1—Pb2	26.76 (11)	C13—N5—N6—O5	171.8 (4)
O8—Pb2—O1—N2	130.8 (3)	Pb2—O5—N6—N5	−29.0 (4)
O5—Pb2—O1—N2	175.6 (3)	Pb1—O5—N6—N5	111.0 (4)
O6—Pb2—O1—N2	−145.0 (3)	Pb2—O8—N7—N8	−8.9 (6)
O7—Pb2—O1—N2	86.3 (3)	Pb2—O8—N7—C19	167.6 (3)
O8—Pb2—O1—Pb1	−75.71 (17)	O8—N7—N8—O7	0.3 (7)
O5—Pb2—O1—Pb1	−30.95 (12)	C19—N7—N8—O7	−176.1 (4)
O6—Pb2—O1—Pb1	8.49 (16)	Pb2—O7—N8—N7	8.3 (5)
O7—Pb2—O1—Pb1	−120.29 (14)	N2—N1—C1—C6	167.7 (4)
O3—Pb1—O2—N1	−81.4 (3)	O2—N1—C1—C6	−11.0 (6)
O4—Pb1—O2—N1	−145.5 (3)	N2—N1—C1—C2	−13.4 (6)
O1—Pb1—O2—N1	−5.8 (3)	O2—N1—C1—C2	168.0 (4)
O5—Pb1—O2—N1	−11.3 (3)	C6—C1—C2—C3	−1.7 (7)
O2—Pb1—O3—N4	−92.5 (3)	N1—C1—C2—C3	179.3 (4)
O4—Pb1—O3—N4	−0.7 (3)	C1—C2—C3—C4	−0.4 (7)
O1—Pb1—O3—N4	−155.7 (3)	C2—C3—C4—C5	1.6 (7)
O5—Pb1—O3—N4	141.1 (4)	C3—C4—C5—C6	−0.7 (7)
O2—Pb1—O4—N3	90.9 (3)	C2—C1—C6—C5	2.6 (7)
O3—Pb1—O4—N3	0.4 (3)	N1—C1—C6—C5	−178.5 (4)
O1—Pb1—O4—N3	35.8 (3)	C4—C5—C6—C1	−1.3 (7)
O5—Pb1—O4—N3	−44.6 (3)	N4—N3—C7—C12	160.2 (4)
O8—Pb2—O5—N6	−42.0 (3)	O4—N3—C7—C12	−21.1 (6)
O6—Pb2—O5—N6	31.3 (3)	N4—N3—C7—C8	−22.9 (6)
O7—Pb2—O5—N6	−106.2 (3)	O4—N3—C7—C8	155.8 (4)
O1—Pb2—O5—N6	171.0 (3)	C12—C7—C8—C9	0.2 (7)
O8—Pb2—O5—Pb1	174.02 (15)	N3—C7—C8—C9	−176.6 (4)
O6—Pb2—O5—Pb1	−112.72 (16)	C7—C8—C9—C10	0.8 (7)
O7—Pb2—O5—Pb1	109.80 (14)	C8—C9—C10—C11	−1.6 (8)
O1—Pb2—O5—Pb1	26.99 (11)	C9—C10—C11—C12	1.4 (8)
O2—Pb1—O5—N6	−166.4 (3)	C8—C7—C12—C11	−0.3 (7)
O3—Pb1—O5—N6	−87.0 (3)	N3—C7—C12—C11	176.5 (4)
O4—Pb1—O5—N6	−45.4 (3)	C10—C11—C12—C7	−0.5 (7)
O1—Pb1—O5—N6	−172.1 (3)	N6—N5—C13—C14	14.3 (6)
O2—Pb1—O5—Pb2	−25.19 (19)	O6—N5—C13—C14	−172.6 (4)
O3—Pb1—O5—Pb2	54.25 (14)	N6—N5—C13—C18	−162.4 (4)
O4—Pb1—O5—Pb2	95.90 (15)	O6—N5—C13—C18	10.7 (6)
O1—Pb1—O5—Pb2	−30.82 (12)	C18—C13—C14—C15	−0.3 (7)
O8—Pb2—O6—N5	59.4 (3)	N5—C13—C14—C15	−176.8 (4)
O5—Pb2—O6—N5	−28.6 (3)	C13—C14—C15—C16	0.2 (7)
O7—Pb2—O6—N5	31.2 (3)	C14—C15—C16—C17	−0.4 (8)
O1—Pb2—O6—N5	−67.2 (3)	C15—C16—C17—C18	0.6 (7)
O8—Pb2—O7—N8	−9.1 (3)	C14—C13—C18—C17	0.5 (7)
O5—Pb2—O7—N8	71.9 (3)	N5—C13—C18—C17	177.1 (4)
O6—Pb2—O7—N8	20.7 (4)	C16—C17—C18—C13	−0.7 (7)
O1—Pb2—O7—N8	134.4 (3)	N8—N7—C19—C24	146.6 (5)
O5—Pb2—O8—N7	−81.3 (3)	O8—N7—C19—C24	−30.1 (6)
O6—Pb2—O8—N7	−147.2 (3)	N8—N7—C19—C20	−33.5 (6)
O7—Pb2—O8—N7	8.6 (3)	O8—N7—C19—C20	149.8 (4)
O1—Pb2—O8—N7	−42.1 (3)	C24—C19—C20—C21	−2.3 (7)
Pb1—O2—N1—N2	6.9 (5)	N7—C19—C20—C21	177.8 (4)
Pb1—O2—N1—C1	−174.5 (3)	C19—C20—C21—C22	0.6 (8)
O2—N1—N2—O1	−1.9 (6)	C20—C21—C22—C23	1.2 (8)
C1—N1—N2—O1	179.6 (4)	C21—C22—C23—C24	−1.4 (8)
Pb1—O1—N2—N1	−4.3 (5)	C22—C23—C24—C19	−0.2 (8)
Pb2—O1—N2—N1	146.7 (3)	C20—C19—C24—C23	2.1 (7)
Pb1—O4—N3—N4	−0.2 (5)	N7—C19—C24—C23	−178.1 (4)
